# Embolia cutis Medicamentosa (Nicolau syndrome): case series

**DOI:** 10.3389/fmed.2023.1216781

**Published:** 2023-11-02

**Authors:** Gyula Laszlo Fekete, Laszlo Barna Iantovics, Júlia Edit Fekete, Laszlo Fekete

**Affiliations:** ^1^Department of Dermatology, George Emil Palade University of Medicine, Pharmacy, Science and Technology of Targu Mures, Targu Mures, Romania; ^2^CMI Dermamed Private Medical Office, Targu Mures, Romania; ^3^Department of Electrical Engineering and Information Technology, George Emil Palade University of Medicine, Pharmacy, Science and Technology of Targu Mures, Targu Mures, Romania; ^4^National Institute of Public Health, Regional Center for Public Health, Targu Mures, Romania; ^5^Doctoral School, George Emil Palade University of Medicine, Pharmacy, Science and Technology of Targu Mures, Targu Mures, Romania

**Keywords:** adverse drug reaction, Nicolau syndrome, embolia cutis, cutaneous gangrene, rare drug reaction

## Abstract

**Introduction:**

Embolia cutis medicamentosa or Nicolau syndrome is a rare drug reaction associated with the administration of various injectable medications. The pathogenesis of the disease is unknown, though intra and periarterial injection of the drug is a possible cause. The aim of this study was to describe and analyze the clinical characteristics of Nicolau syndrome in patients examined in daily dermatological practice.

**Methods:**

We performed a retrospective chart review, between January 2011 and December 2020, in patients diagnosed with Nicolau syndrome, from the cases of a private dermatology medical office in Târgu Mureș, Romania.

**Results:**

During the 10-year period, 7 patients were diagnosed with Nicolau syndrome. Of these, 4 (57%) patients were males and 3 (43%) were females, The male to female ratio was 1.33. The median age was 64 (interquartile range, IQR, 62–71), with the youngest patient being diagnosed at age 61 and the oldest at age 74. Regarding the drugs classes that caused Nicolau syndrome, these were intravenous antibiotics in 57%, and non-steroidal anti-inflammatory drugs in 43% of cases.

**Conclusion:**

All patients healed in a period of 6 to 8 weeks. No complications occurred. In conclusion, Nicolau syndrome is a rare side effect of injectable drug administration.

## 1. Introduction

Nicolau syndrome was first described in the early 1920s by Nicolau as an adverse effect of using intramuscular injections of bismuth salts in the treatment of syphilis (1). Since then, several case reports of this disease occurring after intramuscular, intra-articular, intravenous, and subcutaneous injections, especially in an oily or suspension form, have appeared in the literature associated with a large variety of drugs (2). The pathogenesis of the disease is unknown, though intra and periarterial injection of the drug is a possible cause (3). Stimulation of the sympathetic nerve due to periarterial injection causes spasms and consequent ischemia. Inadvertent intra-arterial injections may cause artery and branch embolization and occlusion, associated with artery wall irritation. Lipophilic drugs can produce fat embolization and cytotoxic drugs may produce inflammation with tissue necrosis. An acute ischemia syndrome of the segmental skin area can occur. The extent and severity of the lesions are closely related to the size of the affected artery. Necrosis ensues in this stage, with possible ulceration (4, 5).

The purpose of this retrospective review was to investigate and chronicle the clinical and disease progression of Nicolau syndrome in patients encountered during routine practice in order to uncover shared characteristics that might foster the medical understanding of the disease.

## 2. Materials and methods

We performed a retrospective chart review between January 2011 and December 2020, in patients diagnosed with Nicolau syndrome, from the cases of a private dermatology medical office in Târgu Mureș, Romania. All patients were Caucasian. Written informed consent of the patients was obtained at the moment of consultation. When making the decision to write this paper, some of the patients were personally invited to the office, and others were contacted by phone to have the study explained and reconfirm the informed consent on the processing of patient personal data. The diagnosis was established by dermatological clinical examination. One investigator evaluated patients and collected data (general data, disease onset, clinical aspect, and evolution, relevant personal history, and present comorbidities). Patients were followed-up during treatment until healing by clinical examination and /or telephone interview. Other investigators, who were blinded to the clinical cases, performed data analysis and interpreted the results.

## 3. Results

Table 1 presents the history and clinical findings. During the 10-year period, 7 patients were diagnosed with Nicolau syndrome. From these, 4 (57%) patients were males and 3 (43%) were females. The male to female ratio was 1.33. The median age was 64 (IQR 62–71) years, with the youngest patient being diagnosed at age 61 and the oldest at age 74.

**Table 1:** Anamnestic and clinical findings.

Case	Age	Gender	Onset of disease	Clinical aspect/ Localization/ Size	Healing	Comorbidities	Incriminated drug
1	74	Female	24 h	Round-oval, anterior forearm right/ 8 cm	8 weeks	Multiple, cardiac and metabolic	Ciprifloxacin 400 mg, iv.
2	71	Female	36 h	Round-oval, anterior forearm right/ 6 cm	6 weeks	Chronic leg ulcer, Diabetes	Cefuroxim 1 g, iv.
3	69	Female	24 h	Dorsal area fist/ 6 cm	6 weeks	Discopathia, Lumbago	Diclofenac 75 mg, iv.
4	62	Male	36 h	Rectangular anterior forearm right/2×6 cm	6 weeks	Chronic leg ulcer, Diabetes	Cefuroxim 1 g, iv.
5	64	Male	36 h	Dorsal area fist/ 5 cm	8 weeks	Chronic leg ulcer, Coxartrozis	Diclofenac 75 mg, iv.
6	61	Male	24 h	Round-oval, anterior forearm right/ 6 cm	6 weeks	Osteolistezis, Leg ulcer, Diabetes	Diclofenac 75 mg, iv.
7	62	Male	24 h	Dorsal area fist/ 5 cm	8 weeks	Chronic leg ulcer, Diabetes	Cefuroxim 1 g, iv

**Figure 1:**
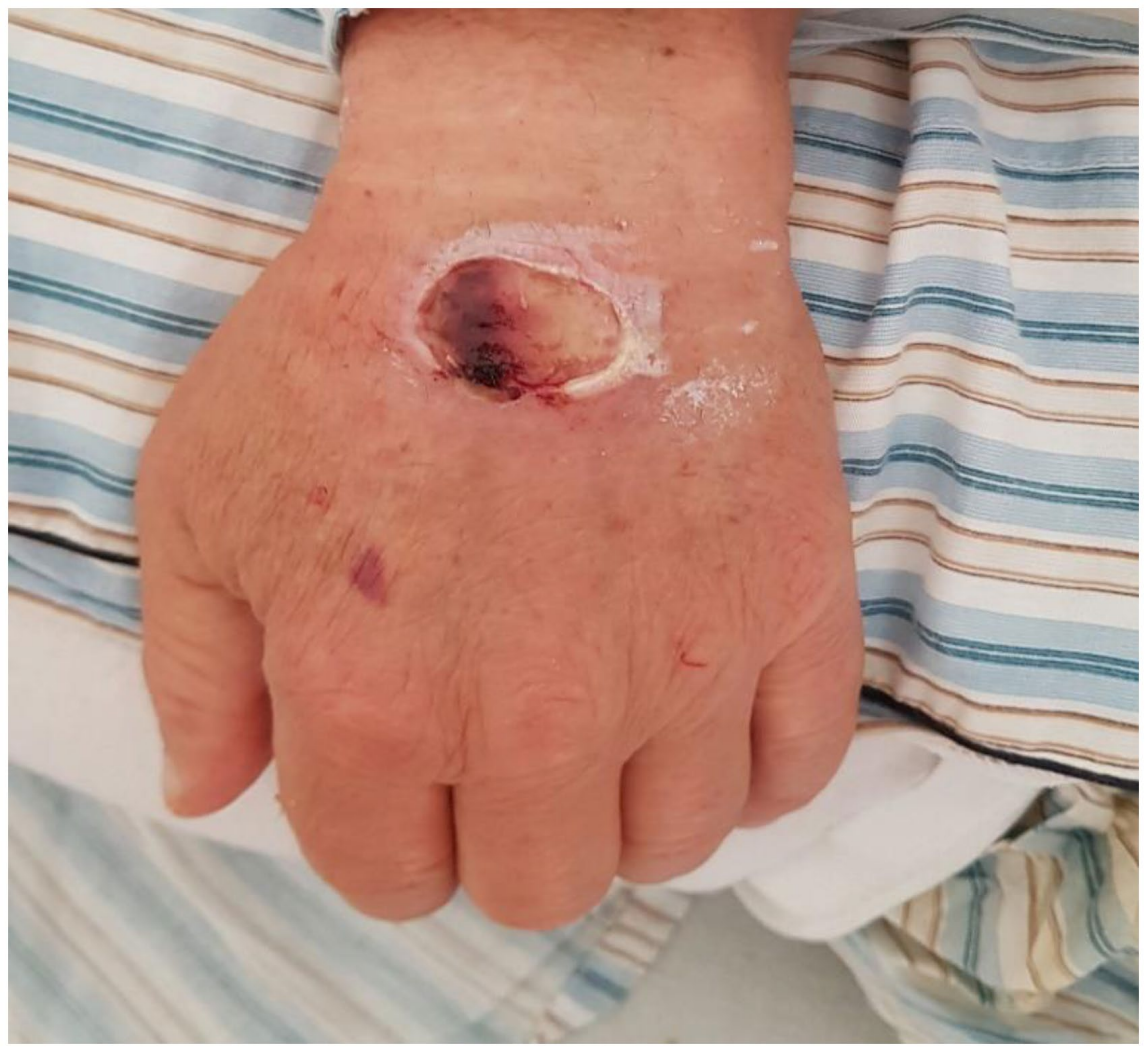
A case of Nicolau syndrome showing a well-defined, oval ulcer with necrotic base on the dorsum of the left hand.

The disease manifested at a median of 24 (IQR 24–36) hours after injection. In 4 out of 7 cases, the lesions appeared within 24 h, while in 3 cases they appeared 36 h after injection. In 4 cases, the lesions were situated on the anterior area of the forearm, the rest on the dorsal area of the fist. Regarding the clinical appearance of the lesions, all of them were red-purple plaques, between 5 and 8 cm in diameter, with a livedoid aspect, centrally necrotic, very painful, with well-defined edges and geographic contours. The lesions were round-oval, apart from one case in which they were rectangular, after using an intravenous branula (Figure 1).

For the verification of outliers, we have applied the Grubbs test (6, 7), which did not detect any outlier.

For all patients the treatment was topical, and consisted of the use of antibiotics and epithelializing ointments, with good results. In some cases, however, surgical debridement was necessary. All patients recovered within a period of 6 to 8 weeks. No complications occurred. All patients suffered from multiple morbid conditions, which was the reason for prescribing injectable, intravenous treatments. Regarding the drugs that caused Nicolau syndrome, in 4 cases it was intravenous antibiotics, and in 3 cases non-steroidal anti-inflammatory drugs. In the group of antibiotics, we found 3 cases of Cefuroxim 1 g as a causative agent 2x1g /day, used intravenously, and in one case Ciprofloxacin 400 mg, 2×400 mg/ day intravenously. Regarding the group of non-steroidal anti-inflammatory drugs in all the 3 cases, the drug was Diclofenac 75 mg, used intravenously (Table 1).

Table 2, row labeled “time to resolution,” presents a descriptive statistic of the healing time. For the verification of outliers, we used the Grubbs test, which did not detect any outlier.

**Table 2:** Descriptive statistic of the onset of the disease.

Characteristics	Time
Latency [h]	24 (24–36)
Time to resolution [d]	42 (42–56)

Continuous data is reported as median and interquartile range.

## 4. Discussions and conclusion

Embolia cutis medicamentosa or Nicolau syndrome is a rare drug reaction associated with the administration of various injectable medications. It is a rare disease, and the true incidence is unknown. The disease can occur at any age, depending on the need to administer intravenous, muscular, or intra-arterial drugs, being linked to the presence of severe comorbidity. The patient’s data analyzed in this study was carefully collected and recorded in a database during the 10-year period. Over this time, we detected 7 patients, 4 males and 3 females, median age 64 years, having suffered from Nicolau syndrome.

Mojjarad et al., analyzing 135 cases from multiple databases on Nicolau syndrome and concluded that the disease can occur mainly in females of any age group, but mainly in children and the age group 31–40 years (8). Regarding the onset of the disease due to the appearance of pain at the level of injection, in our cases, the latency was between 24 and 36 h, which corresponds to the data from meta-analytical studies (8). The clinical appearance of the skin lesions is identical in each case, regardless of the cause, the site of injection being a single round oval lesion with a diameter between 5 and 8 cm, well delimited, with ulceration and necrosis on the surface. In our retrospective case series, the healing period was between 6 and 8 weeks, regardless of the causative agent or location. The prescribed treatments were similar in these cases and the disease course was favorable. Local treatments included antibiotics, epithelisants, magistral prescriptions with Silver nitrate, and Peru Balsam used in difficult-to-heal ulcers (9). In our cases, previously reported complications such as fasciitis, superinfections, amputations, or deaths did not occur (10–13). A multitude of drugs can cause this disease, among which the most important groups of drugs are non-steroidal anti-inflammatory drugs and antibiotics. In a review assessing 145 articles, Mojjarad et al. found that the most common causes of the syndrome are diclofenac (35 articles, 24%) and penicillins (32 articles, 22%) (8). In our cases, the cause of the injection was Diclofenac in 3 patients; Cefuroxime in 3 patients and Ciprofloxacin in 1 patient. According to previously published literature, Gentamicin (14) and Penicillins (15, 16) are among the most frequently reported antibiotics causing Nicolau syndrome. Among the non-steroidal anti-inflammatory drugs, Diclofenac is the most frequently reported drug, followed by other drugs such as Naltroxene, Etofenamate, and Ketofrofenid (17–20). Cases of Nicolau syndrome caused by dermatocosmetic procedures such as hyaluronic acid injections (21), meso, and sclerotherapy (22, 23) have been described. Other drug classes have been mentioned as causative factors such as: triamcinolone (24), glatiramer acetate (25), terlipressin (26), bortezomib (27), hydroxyzine (28), interferon alfa (29), etc. At the same time, it may occur as a local reaction after the administration of vaccines such as the hexavalent vaccine (30), or the DTP vaccine (31). In conclusion, Nicolau syndrome is a rare side effect of injectable drugs which can be severe.

## Data availability statement

The raw data supporting the conclusions of this article will be made available by the authors, without undue reservation.

## Ethics statement

The studies involving humans were approved by Dermamed private office ethics committee. The studies were conducted in accordance with the local legislation and institutional requirements. The participants provided their written informed consent to participate in this study. Written informed consent was obtained from the participant/patient(s) for the publication of this article.

## Author contributions

GF, LF, and JF: conceptualization, methodology, validation, formal analysis, investigation, and writing—review and editing. LI: software, data curation, and writing—original draft preparation. GF and LF: resources. GF, LF, JF, and LI: visualization. GF: supervision, project administration and funding acquisition. All authors contributed to the article and approved the submitted version.
